# Effect of preoperative alpha‐blockers on ureteroscopy outcomes: A meta‐analysis of randomised trials

**DOI:** 10.1002/bco2.358

**Published:** 2024-04-03

**Authors:** Naeem Bhojani, Ben H. Chew, Samir Bhattacharyya, Amy E. Krambeck, Khurshid R. Ghani, Larry E. Miller

**Affiliations:** ^1^ Division of Urology Centre Hospitalier de l'Université de Montréal, Montréal Québec Canada; ^2^ Department of Urologic Sciences University of British Columbia Vancouver BC Canada; ^3^ Health Economics and Market Access Boston Scientific Marlborough Massachusetts USA; ^4^ Department of Urology Northwestern University School of Medicine Chicago Illinois USA; ^5^ Department of Urology University of Michigan Medical School Ann Arbor Michigan USA; ^6^ Department of Biostatistics Miller Scientific Johnson City Tennessee USA

**Keywords:** alpha‐blocker, kidney stone, silodosin, tamsulosin, ureteral, ureteroscopy

## Abstract

**Objectives:**

This work aims to determine the efficacy and safety of preoperative alpha‐blocker therapy on ureteroscopy (URS) outcomes.

**Methods:**

In this systematic review and meta‐analysis of randomised trials of URS with or without preoperative alpha‐blocker therapy, outcomes included the need for ureteral dilatation, stone access failure, procedure time, residual stone rate, hospital stay, and complications. Residual stone rates were reported with and without adjustments for spontaneous stone passage, medication noncompliance, or adverse events leading to patient withdrawal. Data were analysed using random‐effects meta‐analysis and meta‐regression. Certainty of evidence was assessed using the GRADE criteria.

**Results:**

Among 15 randomised trials with 1653 patients, URS was effective and safe with a stone‐free rate of 81.2% and rare (2.3%) serious complications. The addition of preoperative alpha‐blockers reduced the need for ureteral dilatation (risk ratio [RR] = 0.48; 95% CI = 0.30 to 0.75; *p* = 0.002), access failure rate (RR = 0.36; 95% CI = 0.23 to 0.57; *p* < 0.001), procedure time (mean difference [MD] = −6 min; 95% CI = −8 to −3; *p* < 0.001), risk of residual stone in the primary (RR = 0.44; 95% CI = 0.33 to 0.66; *p* < 0.001) and adjusted (RR = 0.52; 95% CI = 0.40 to 0.68; *p* < 0.001) analyses, hospital stay (MD = −0.3 days; 95% CI = −0.4 to −0.1; *p* < 0.001), and complication rate (RR = 0.46; 95% CI = 0.35 to 0.59; *p* < 0.001). Alpha‐blockers increased ejaculatory dysfunction risk and were less effective for renal/proximal ureter stones. The certainty of evidence was high or moderate for all outcomes. The main limitation of the review was inconsistency in residual stone assessment methods.

**Conclusion:**

While URS is an effective and safe treatment for stone disease, preoperative alpha‐blocker therapy is well tolerated and can further improve patient outcomes.

## 1 INTRODUCTION

Kidney stone disease is responsible for a significant clinical and economic burden among US adults, affecting 11% of adults with annual medical costs exceeding $10 billion.^1^ Patients with symptomatic ureteral stones ≤10 mm are typically managed with pain control, medical expulsive therapy (MET), and serial imaging to monitor stone position and assess for hydronephrosis. However, spontaneous passage of larger or proximal stones is uncommon. Patients with persistent complications such as pain, nausea, and renal insufficiency are candidates for definitive stone treatment using ureteroscopy (URS), shockwave lithotripsy, or percutaneous nephrolithotomy. With a significant increase in utilisation over the last decade,^2^ URS has become the most common interventional treatment for ureteric and renal stones, achieving stone‐free rates generally ranging from 75% to 85%.^3^, ^4^

To further improve the effectiveness and success of URS, preoperative selective alpha‐1 adrenergic receptor antagonists (alpha‐blockers) may be considered. Selective alpha‐blockers bind to type 1 alpha‐adrenergic receptors, thus inhibiting smooth muscle contraction. Meta‐analyses have reported that alpha‐blockers improve stone clearance rates when used as MET, but may be less effective for proximal stones.^5^, ^6^ Although patients scheduled for URS tend to have stones that are too large or located too proximally for alpha‐blockers to assist in MET, these medications may facilitate stone removal during URS by relaxing ureteral smooth muscles and lowering intramural ureteral resistance, allowing easier insertion of necessary equipment such as a ureteroscope into the ureteral orifice, which may minimise mucosal wall damage. Several systematic reviews of randomised controlled trials (RCTs) have evaluated the utility of preoperative alpha‐blockers on URS outcomes.^7^, ^8^, ^9^ However, no review has attempted to identify determinants of residual stone and complication risk with preoperative alpha‐blocker therapy. Therefore, we performed a systematic review and meta‐analysis of RCTs, hypothesising that preoperative alpha‐blocker therapy compared with control treatment would reduce the risk of residual stones and complications after URS.

## 2 METHODS

The methods, analysis, and reporting of this review followed the Preferred Reporting Items for Systematic Reviews and Meta‐analyses (PRISMA).^10^ We prospectively registered the review protocol and analysis plan at www.researchregistry.com (reviewregistry1577).

### 2.1 Search strategy and study selection

Eligible studies were RCTs of URS for renal or ureteral stone treatment with or without preoperative alpha‐blocker therapy. Randomised controlled trials were selected because they provide more reliable estimates of treatment effects than nonrandomised studies by minimising bias and confounding factors. We systematically searched Medline, Embase, and the Cochrane Central Register of Controlled Trials for potentially eligible studies, with no date or language restrictions. In addition, supplemental manual searches were conducted in the Directory of Open Access Journals, Google Scholar, and the reference lists of included papers and relevant meta‐analyses. The search strategy (Table S1) utilised combinations of medication‐ and procedure‐related search terms. Two researchers (LM, DF) independently screened titles and abstracts for eligibility. Studies involving children (under 18 years), combination medical therapy, or those published only as abstracts or presentations were excluded. Full‐text manuscripts were obtained for all eligible studies and those with uncertain eligibility. Disagreements related to study eligibility were resolved by discussion. The last searches were performed in April 2023.

### 2.2 Data extraction and outcomes

Two researchers (LM, DF) independently recorded data from included studies on standardised data collection forms, and disagreements were resolved by discussion. Key data elements were study metadata, patient characteristics, study characteristics, treatment regimens, and main outcomes. The main outcomes were the need for ureteral dilatation, stone access failure, procedure time, residual stone risk, length of hospital stay, and complications. We analysed unadjusted and adjusted residual stone rates, with adjustments for spontaneous stone passage (counted as a success) and medication noncompliance or adverse events leading to patient withdrawal before URS (counted as failures). For studies that reported residual stone rates at multiple time points, we extracted data at the interval closest to 4 weeks after URS. Complications were reported as the proportion of patients with any complication and the proportion with a serious (Clavien‐Dindo grade III‐V) complication.

### 2.3 Risk of bias assessment

We used the Cochrane Collaboration tool to assess the risk of bias in individual studies.^11^ Individual studies were evaluated for selection, performance, detection, attrition, reporting, and other potential sources of bias. In addition, the risk of bias was summarised across studies and bias domains.

### 2.4 Statistical analysis

Data were analysed using a random‐effects meta‐analysis model. In accordance with Cochrane recommendations,^12^ multiple groups from the same study were pooled to create a single pairwise comparison, thus avoiding double‐counting of control patients. Outcomes reported on a continuous scale were analysed using the mean difference (MD) statistic. Negative MD values indicated better results in the alpha‐blocker group, and positive MD values indicated better results in the control group. Binary outcomes were reported using the risk ratio (RR) statistic. A RR < 1 indicated lower risk with alpha‐blockers, and a RR > 1 indicated higher risk with alpha‐blockers. We displayed the effect size and 95% confidence interval (CI) for each outcome using forest plots. We reported heterogeneity among studies with the *I* ^2^ statistic, where 0% indicated no heterogeneity and larger values represented increasing heterogeneity.^13^ Significant heterogeneity was defined by *I* ^2^ > 50%. We visually inspected funnel plots for potential publication bias. We planned to perform a meta‐regression on the association of study‐level factors with the risk of residual stones and complications. *p* values were two‐sided with a significance level <0.05. Analyses were performed by an independent biostatistician using Review Manager v5.4 (The Cochrane Collaboration).

### 2.5 GRADE certainty of evidence

We used the Grading of Recommendations Assessment, Development and Evaluation (GRADE) criteria to assess the certainty of evidence.^14^

## 3 RESULTS

The systematic review identified 15 RCTs^15^, ^16^, ^17^, ^18^, ^19^, ^20^, ^21^, ^22^, ^23^, ^24^, ^25^, ^26^, ^27^, ^28^, ^29^ with 1653 patients (844 alpha‐blockers; 809 controls) included in the meta‐analysis (Figure S1). Preoperative alpha‐blocker therapy involved tamsulosin (11 studies) or silodosin (four studies) administered over 1 to 56 days before URS (median 7 days). Controls received placebo (seven studies) or no additional preoperative treatment (eight studies). Patients with a history of endoscopic or open ureteral surgery were routinely excluded from participation in the trials. Stone location and diameter varied considerably among studies (Table S2). A risk of bias summary for each study is provided in Figure S2. The most common risk of bias was related to selective reporting where patients were commonly excluded from the analysis due to adverse events, medication noncompliance, or stone expulsion before URS. We also noted variability in the modalities and definitions used to determine stone‐free status after URS. These were reflected as Other Bias in the risk of bias summary and described in Table S3.

Comparing alpha‐blockers to controls, the percentage of patients excluded from primary analyses among studies was 1.3% versus 0.5% for stone expulsion before URS and 3.8% versus 1.8% for study withdrawal before URS due to medication‐related noncompliance or adverse events. Few studies reported medication‐related adverse event rates in both groups.^20^, ^28^ The only adverse event that differed between groups was a higher risk of ejaculatory dysfunction in the alpha‐blocker group (12.9% vs. 3.2%; *p* = 0.05).

**FIGURE 1:**
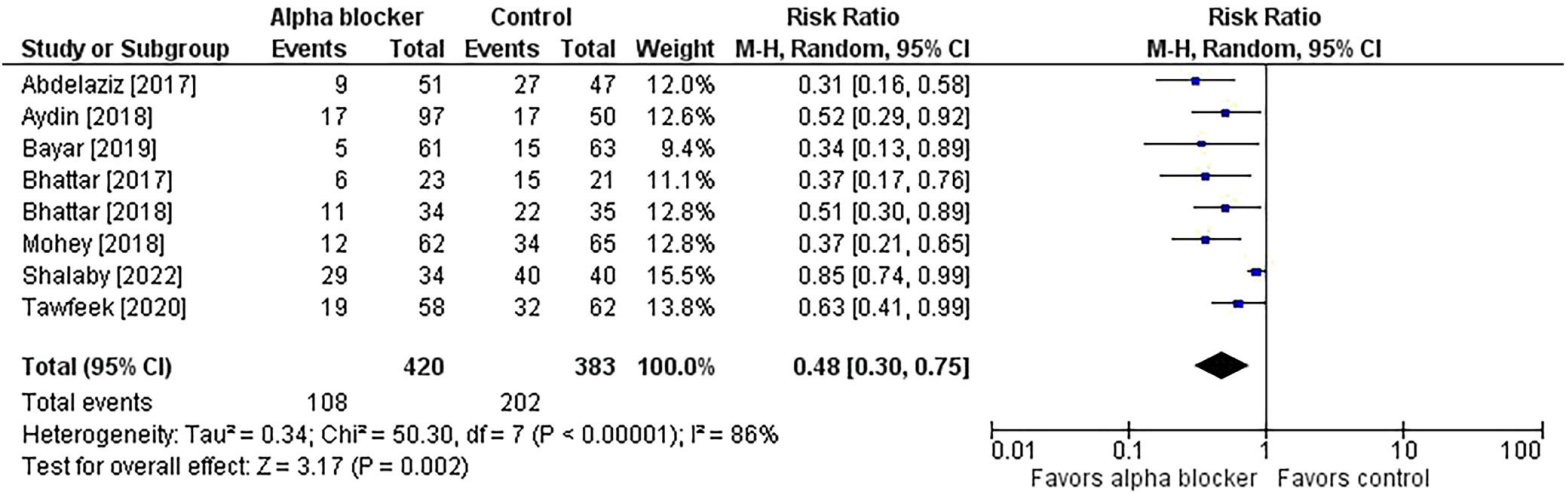
Effect of alpha‐blocker therapy before ureteroscopy on the risk of requiring ureteral dilatation. The risk ratio and 95% confidence interval are plotted for each study. The pooled risk ratio (diamond apex) and 95% confidence interval (diamond width) are calculated using a random effects model. Abbreviations: CI = confidence interval; M‐H = Mantel–Haenszel.

**FIGURE 2:**
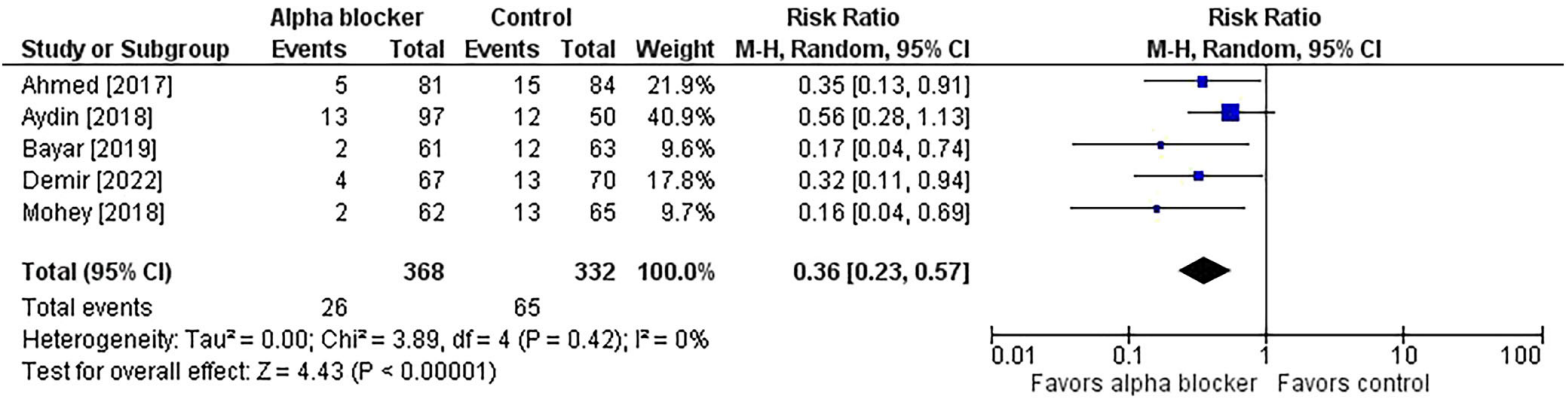
Effect of alpha‐blocker therapy before ureteroscopy on the risk of stone access failure. The risk ratio and 95% confidence interval are plotted for each study. The pooled risk ratio (diamond apex) and 95% confidence interval (diamond width) are calculated using a random effects model. Abbreviations: CI = confidence interval; M‐H = Mantel–Haenszel.

**FIGURE 3:**
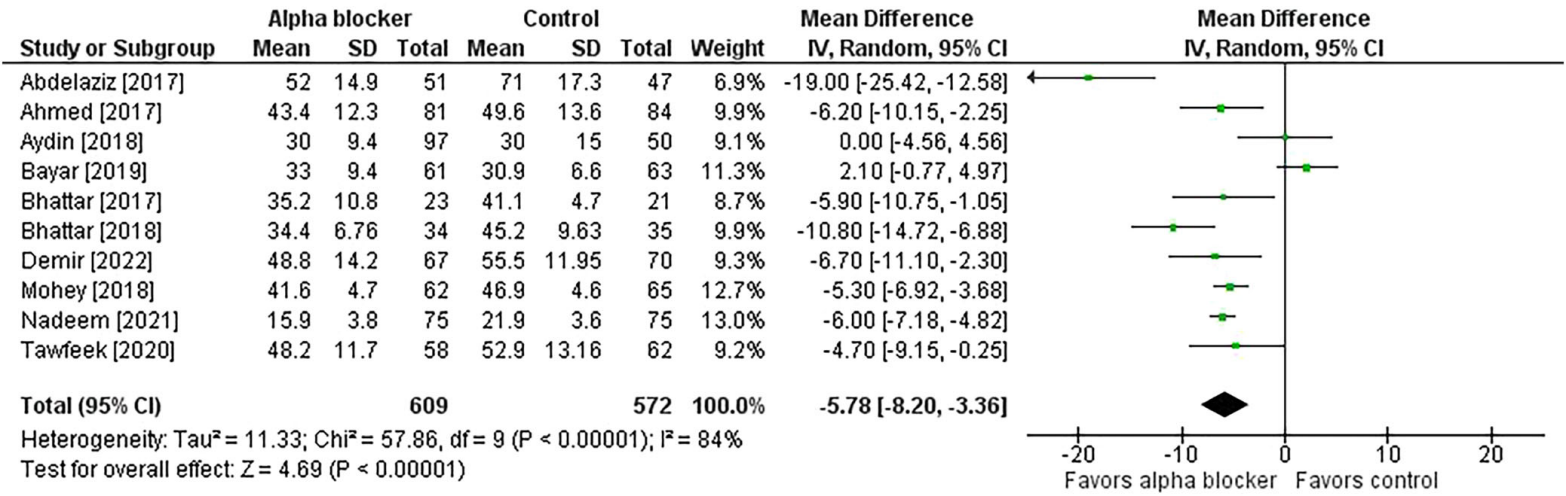
Effect of alpha‐blocker therapy before ureteroscopy on procedure time. Values reported in minutes. The mean difference and 95% confidence interval are plotted for each study. The pooled mean difference (diamond apex) and 95% confidence interval (diamond width) are calculated using a random effects model. Abbreviations: CI = confidence interval; IV = inverse variance; SD = standard deviation.

**FIGURE 4:**
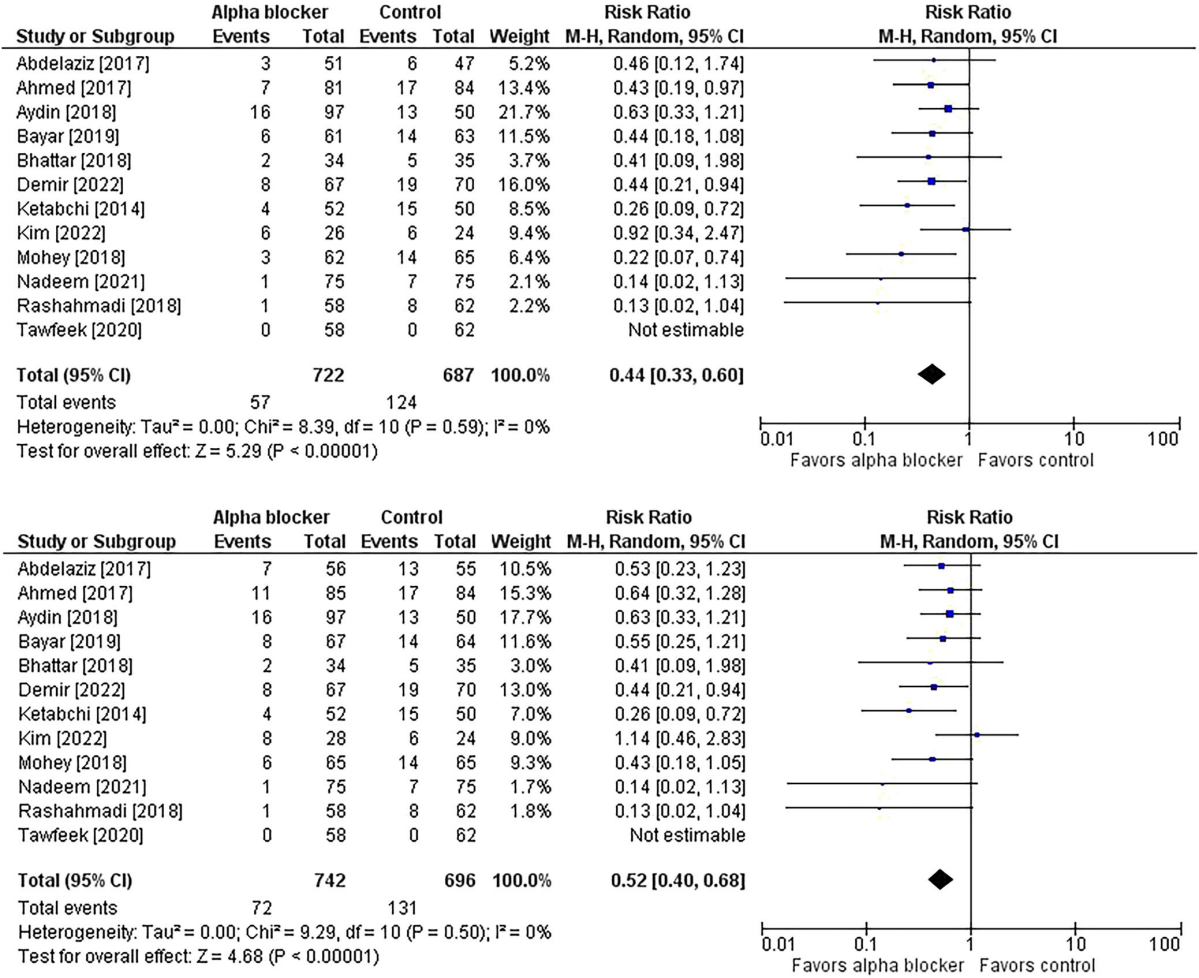
Effect of alpha‐blocker therapy before ureteroscopy on the risk of residual stone. (top panel) Results as reported within studies; (bottom panel) Sensitivity analysis with results adjusted for pre‐treatment stone expulsion and study withdrawals due to medication non‐compliance or adverse events. The risk ratio and 95% confidence interval are plotted for each study. The pooled risk ratio (diamond apex) and 95% confidence interval (diamond width) are calculated using a random effects model. Abbreviations: CI = confidence interval; M‐H = Mantel–Haenszel.

**FIGURE 5:**
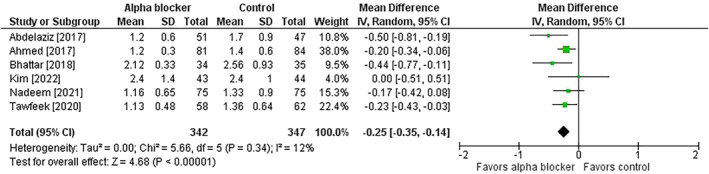
Effect of alpha‐blocker therapy before ureteroscopy on hospital stay. Values reported in days. The mean difference and 95% confidence interval are plotted for each study. The pooled mean difference (diamond apex) and 95% confidence interval (diamond width) are calculated using a random effects model. Abbreviations: CI = confidence interval; IV = inverse variance; SD = standard deviation.

**FIGURE 6:**
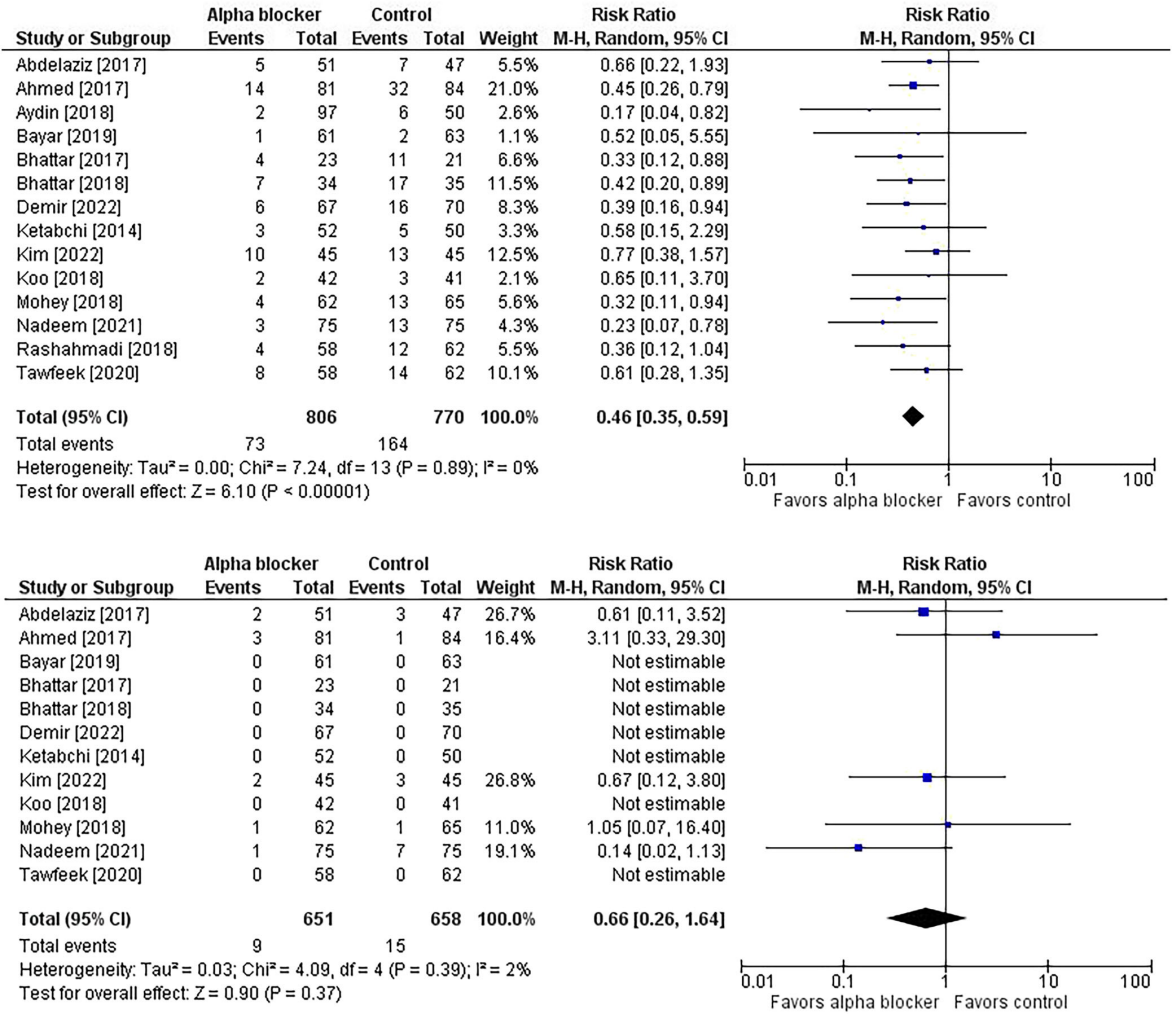
Effect of alpha‐blocker therapy before ureteroscopy on the risk of complications (top) and serious complications (bottom). The risk ratio and 95% confidence interval are plotted for each study. The pooled risk ratio (diamond apex) and 95% confidence interval (diamond width) are calculated using a random effects model. Abbreviations: CI = confidence interval; M‐H = Mantel–Haenszel.

Preoperative alpha‐blocker therapy resulted in statistically significant improvements in all outcomes compared with patients receiving control. Comparing alpha‐blockers versus controls, ureteral dilatation was performed in 25.7% versus 52.7% of patients (risk ratio = 0.48; 95% CI = 0.30 to 0.75; *p* = 0.002; *I* ^2^ = 86%) (Figure 1). Stone access failure rates were 7.1% versus 19.6% (risk ratio = 0.36; 95% CI = 0.23 to 0.57; *p* < 0.001; *I* ^2^ = 0%) (Figure 2). Procedure times were shorter in patients treated with preoperative alpha‐blockers (MD = −6 min; 95% CI = −8 to −3; *p* < 0.001; *I* ^2^ = 84%) (Figure 3). The risk of post‐URS residual stone was lower in patients receiving preoperative alpha‐blocker therapy in the primary (7.9% vs. 18.0%; risk ratio = 0.44; 95% CI = 0.33 to 0.66; *p* < 0.001; *I* ^2^ = 0%) and adjusted analyses (9.7% vs. 18.8%; risk ratio = 0.52; 95% CI = 0.40 to 0.68; *p* < 0.001; *I* ^2^ = 0%) (Figure 4). The length of hospital stay was shorter in the alpha‐blocker group (MD = −0.3 days; 95% CI = −0.4 to −0.1; *p* < 0.001; *I* ^2^ = 12%) (Figure 5); however, in studies with missing data, it was unclear whether hospital stay was unreported or if procedures were performed on an outpatient basis. The risk of complications was lower in patients receiving preoperative alpha‐blocker therapy (9.1% vs. 21.3%; risk ratio = 0.46; 95% CI = 0.35 to 0.59; *p* < 0.001; *I* ^2^ = 0%). Serious complications were rare with no difference in risk between groups (1.4% vs. 2.3%; risk ratio = 0.66; 95% CI = 0.26 to 1.64; *p* = 0.37; *I* ^2^ = 2%) (Figure 6). There was no evidence of publication bias for any outcome (Figures S3–S9). In meta‐regression, alpha‐blockers were less effective in reducing the residual stone risk for renal/proximal ureter stones compared with distal/mid‐ureter stones (Figure S10). No patient‐ or study‐related factor, including stone size or location, modified the effect of alpha‐blocker therapy on the risk of complications (Table S4). The certainty of evidence in this meta‐analysis was rated high or moderate for all outcomes (Table S5).

## 4 DISCUSSION

In this systematic review and meta‐analysis of RCTs, URS was effective and safe for treating stone disease with a stone‐free rate of 81.2% and 21.3% of patients reporting primarily minor complications (Clavien‐Dindo grade I‐II). The addition of preoperative alpha‐blocker therapy improved these rates to 90.3% and 9.1%, respectively. Alpha‐blockers also reduced the need for ureteral dilatation, improved stone access rates, and were associated with shorter procedure times and hospital stays. Although alpha‐blockers were well tolerated, they increased the risk of ejaculatory dysfunction. Also, alpha‐blockers were less effective for renal/proximal ureter stones. Overall, the results of this meta‐analysis suggest that preoperative alpha‐blocker therapy can improve the effectiveness and safety of URS.

The mechanism of action underlying the efficacy of preoperative alpha‐blocker therapy is likely the relaxation of distal ureteric smooth muscle and dilatation of the distal ureteric lumen,^30^ resulting in easier retrograde catheterisation and improved stone access. The finding that alpha‐blockers were more effective for distal ureter stones is supported by the higher concentration of alpha receptors in the distal ureter.^30^ Alpha‐blockers are commonly used for MET and are most effective for smaller and distal stones.^31^ To the authors' knowledge, this review is the first to demonstrate an association between preoperative alpha‐blocker efficacy and stone location.

Alpha‐blockers were well tolerated overall with only 3.8% of patients withdrawing from studies due to medication‐related noncompliance or adverse events. Nonetheless, alpha‐blockers were associated with a higher risk of ejaculatory dysfunction. Uroselective alpha‐blockers relax the tone of smooth muscles in the bladder neck, reducing the pressure proximal to the urethral crest. Although the clinical course of patients reporting ejaculatory dysfunction was not described in these studies, patients usually regain normal ejaculatory function after alpha‐blocker discontinuation.^32^ Selective alpha‐blockers may be inappropriate for patients with orthostatic hypotension because lightheadedness and fainting are known side effects. Also, drug interactions with beta‐blockers, erectile dysfunction drugs, anxiolytics, and antihistamines may exacerbate these effects. Overall, the safety of alpha‐blockers appears acceptable because most associated risks are minor and reversible.

This meta‐analysis provided Level 1 evidence with moderate to high certainty to support alpha‐blocker therapy before URS for treating stone disease. However, there were several important limitations of this review that warrant discussion. First, there was considerable variation in stone size and location among studies, and the reporting of these data was often incomplete. This may have hindered our ability to determine the association of these variables with the risk of residual stones and complications. Second, most RCTs in this review were rated high risk of reporting bias because patients were commonly excluded from primary analysis for spontaneous stone expulsion and medication‐related study withdrawal. We adjusted for this bias in our analysis of residual stone risk. However, the impact of this bias on other study outcomes remains unclear. Third, the diagnosis of postoperative residual stone was made inconsistently, where imaging modalities, percentage of residual stone classified as stone free, and follow‐up duration varied among studies. Fourth, the length of hospital stay was only reported in six of 15 studies. For the studies with missing data, it was unclear whether hospital stay was simply unreported or if procedures were performed in an outpatient setting. Additionally, several other outcomes of this meta‐analysis were reported infrequently among the included studies. This was reflected by a moderate certainty of evidence using the GRADE criteria, and suggests the influence of preoperative alpha‐blocker therapy on these outcomes should be interpreted cautiously. Finally, we could not accurately determine the proportion of patients reporting adverse events before URS due to incomplete reporting among studies. Nonetheless, alpha‐blockers were well tolerated overall given the study dropout rates of 3.8% and 1.8%, respectively, due to medication‐related noncompliance or adverse events. Overall, despite the evidence in favour of preoperative alpha‐blocker therapy, inconsistencies between studies regarding factors like medication type and dosing, outcome definitions, and imaging protocols restrict making more specific recommendations about optimal prescribing regimens.

Notwithstanding the limitations, our study presents a comprehensive systematic review and meta‐analysis of preoperative alpha‐blocker therapy for URS that can assist healthcare providers in evaluating the efficacy of this treatment strategy. Because many patients with ureteral stones awaiting URS have already been prescribed alpha‐blocker therapy for ureteral colic, continued use of this medication may be prudent in these cases because there was no evidence that longer courses of preoperative alpha blockade negatively influenced outcomes. For patients who have not initiated alpha‐blocker therapy, we recommend commencing treatment 1 to 7 days before the scheduled URS, and discontinuing therapy after the procedure. Based on the regimens reviewed, reasonable daily alpha‐blocker dosages are 8 mg of silodosin, or 0.4 to 0.8 mg of tamsulosin. Moreover, it is essential to counsel male patients on the potential risk of ejaculatory dysfunction while on therapy. Overall, this review provides useful insights into the role of preoperative alpha‐blocker therapy for URS that may guide clinical decision‐making in this area.

## 5 CONCLUSIONS

While URS is an effective and safe treatment for stone disease, preoperative alpha‐blocker therapy is well tolerated and can further improve patient outcomes.

## AUTHOR CONTRIBUTIONS

SB and LM conceived and designed the study. LM and DF (acknowledged) performed the database search, study selection, and data extraction. LM conducted the statistical analysis. All authors interpreted data for the work. LM drafted the article. All authors reviewed and revised the article. All authors read and approved the final article.

## CONFLICT OF INTEREST STATEMENT

Drs. Bhojani, Chew, and Krambeck report consultancy with Boston Scientific (unrelated to the current study). Dr. Bhattacharyya reports employment with Boston Scientific. Dr. Ghani reports consultancy with Boston Scientific (unrelated to the current study) and investigator funding from Olympus, Storz, Ambu, and Coloplast. Dr. Miller reports consultancy with Boston Scientific (related to the current study).

## Supporting information


**Table S1.** MEDLINE search strategy^a^
**Table S2.** Patient and study characteristics.
**Table S3.** Definitions and modalities used to determine stone‐free status
**Table S4.** Association of patient‐ and study‐factors on the risk of residual stone and complications with alpha‐blocker therapy before ureteroscopy.*
**Table S5.** Grading of Recommendations Assessment, Development and Evaluation (GRADE) certainty of evidence.
**Figure S1.** PRISMA flow diagram.
**Figure S2.** Risk of bias summary. Review authors' judgements about each risk of bias item for each included study (top) and presented as percentages across all included studies (bottom).
**Figure S3.** Funnel plot of alpha‐blocker therapy before ureteroscopy on the risk of requiring ureteral dilatation. Abbreviations: RR = risk ratio; SE = standard error.
**Figure S4.** Funnel plot of alpha‐blocker therapy before ureteroscopy on the risk of stone access failure. Abbreviations: RR = risk ratio; SE = standard error.
**Figure S5.** Funnel plot of alpha‐blocker therapy before ureteroscopy on procedure time. Values reported in minutes. Abbreviations: MD = mean difference; SE = standard error.
**Figure S6.** Funnel plot of alpha‐blocker therapy before ureteroscopy on the risk of residual stone. Abbreviations: RR = risk ratio; SE = standard error.
**Figure S7.** Funnel plot of alpha‐blocker therapy before ureteroscopy on hospital stay. Values reported in days. Abbreviations: MD = mean difference; SE = standard error.
**Figure S8.** Funnel plot of alpha‐blocker therapy before ureteroscopy on the risk of complications. Abbreviations: RR = risk ratio; SE = standard error.
**Figure S9.** Funnel plot of alpha‐blocker therapy before ureteroscopy on the risk of serious complications. Abbreviations: RR = risk ratio; SE = standard error.
**Figure S10.** Bubble plot of the association between the log risk ratio of residual stones and stone location. Open circles represent values of individual studies where the circle size is proportional to the study weight in the random‐effects model. The red line represents the regression line of best fit. A log risk ratio (logRR) value of 0 indicates no effect of alpha‐blockers; negative values indicate lower risk of residual stone with alpha‐blockers.

## Data Availability

The data from this review are available upon reasonable request.
