# Supplementary material for: Effect of preoperative alpha‐blockers on ureteroscopy outcomes: A meta‐analysis of randomised trials
Source: BJUI Compass. 2024 Apr 3;5(7):613–20. doi: 10.1002/bco2.358 (PMC11249831; doi:10.1002/bco2.358)
Supplement: Supplementary file 1 — Table S1. MEDLINE search strategya Table S2. Patient and study characteristics. Table S3. Definitions and modalities used to determine stone‐free status Table S4. Association of patient‐ and study‐factors on the risk of residual stone and complications with alpha‐blocker therapy before ureteroscopy.* Table S5. Grading of Recommendations Assessment, Development and Evaluation (GRADE) certainty of evidence. Figure S1. PRISMA flow diagram. Figure S2. Risk of bias summary. Review authors' judgements about each risk of bias item for each included study (top) and presented as percentages across all included studies (bottom). Figure S3. Funnel plot of alpha‐blocker therapy before ureteroscopy on the risk of requiring ureteral dilatation. Abbreviations: RR = risk ratio; SE = standard error. Figure S4. Funnel plot of alpha‐blocker therapy before ureteroscopy on the risk of stone access failure. Abbreviations: RR = risk ratio; SE = standard error. Figure S5. Funnel plot of alpha‐blocker therapy before ureteroscopy on procedure time. Values reported in minutes. Abbreviations: MD = mean difference; SE = standard error. Figure S6. Funnel plot of alpha‐blocker therapy before ureteroscopy on the risk of residual stone. Abbreviations: RR = risk ratio; SE = standard error. Figure S7. Funnel plot of alpha‐blocker therapy before ureteroscopy on hospital stay. Values reported in days. Abbreviations: MD = mean difference; SE = standard error. Figure S8. Funnel plot of alpha‐blocker therapy before ureteroscopy on the risk of complications. Abbreviations: RR = risk ratio; SE = standard error. Figure S9. Funnel plot of alpha‐blocker therapy before ureteroscopy on the risk of serious complications. Abbreviations: RR = risk ratio; SE = standard error. Figure S10. Bubble plot of the association between the log risk ratio of residual stones and stone location. Open circles represent values of individual studies where the circle size is proportional to the study weight in the random‐effects mod [file BCO2-5-613-s001.docx]

**Supporting Information**

**Table S1. MEDLINE search strategy^a^**

| Medication Search Terms | |
| --- | --- |
| 1. Alfuzosin 2. Alpha-1 antagonist 3. Alpha-1 blocker 4. Alpha-adrenergic antagonist 5. Alpha blocker 6. Doxazosin 7. Prazosin 8. Silodosin 9. Tamsulosin 10. Terazosin | |
| Procedure Search Terms | |
| 1. Retrograde intrarenal surgery 2. RIRS 3. Ureterorenoscop* 4. Ureteroscop* | |
| Combination Terms | |
| 1. or/1-10 2. or/11-14 | |
| 1. and/15-16 | |

^a^The ‘*’ represents a wildcard symbol used in a search query to represent end truncation.

**Table S2. Patient and study characteristics.**

| **Study** | **Treatment**  **period** | **Sample**  **Size *** | **Mean**  **Age *** | **Male**  **sex (%) *** | **Active**  **medication** | **Control**  **medication** | **Pre-URS**  **medication**  **duration** | **Post-URS**  **medication duration** | **Stone**  **location** | **Stone**  **diameter** | **Number of stones** |
| --- | --- | --- | --- | --- | --- | --- | --- | --- | --- | --- | --- |
| Abdelaziz [2017] (15) | 2011-2014 | 51 / 47 | 35 / 38 | 67 / 64 | Tamsulosin, 0.4 mg qd | None | 7 days | None | Distal ureter | 5-10 mm  (mean 6.4 mm) | Single |
| Ahmed [2017] (16) | 2013-2015 | 81 / 84 | 36 / 38 | 62 / 57 | Tamsulosin, 0.4 mg qd | None | 7 days | None | Proximal ureter | 10-15 mm  (mean 13.0 mm) | Single |
| Aydin [2018] (17) | 2016-2016 | 97 / 50 ** | 80 / 38 | 72 / 66 | Silodosin,  8 mg qd | None | 1 or 3 days | None | Ureter (varied) | ≤ 20 mm  (mean 6.4 mm) | Single |
| Bayar [2019] (18) | 2017-2018 | 61 / 63 | 42 / 39 | 69 / 67 | Tamsulosin, 0.4 mg qd | None | 7 days | None | Ureter (varied) | ≤ 20 mm  (mean 7.4 mm) | Single |
| Bhattar [2017] (19) | 2015-2016 | 23 / 21 | 35 / 33 | 65 / 71 | Silodosin,  8 mg qd | Placebo,  qd | 14 days | None | Ureter (varied) | 6-15 mm  (mean 9.4 mm) | Single |
| Bhattar [2018] (20) | 2016-2017 | 34 / 35 | 35 / 37 | 71 / 71 | Tamsulosin, 0.4 mg qd | Placebo,  qd | 10 days | None | Distal/mid ureter | 8-15 mm  (mean 10.7 mm) | Single |
| Demir [2022] (21) | 2020-2021 | 67 / 70 | 47 / 46 | 75 / 75 *** | Tamsulosin, 0.4 mg qd | None | 7 days | None | Ureter (varied) | ≥ 7 mm  (mean 11.5 mm) | Single |
| Ketabchi [2014] (22) | 2008-2010 | 52 / 50 | 24 / 27 | 71 / 80 | Tamsulosin, 0.4 mg qd | Placebo,  qd | 1 day | None | Distal ureter | 5-10 mm  (mean 6.4 mm) | Single |
| Kim [2022] (23) | 2018-2019 | 43 / 44 | 48 / 46 | 67 / 52 | Silodosin,  8 mg qd | None | 3 days | None | Proximal ureter; renal | < 20 mm  (mean 8.8 mm) | Single |
| Koo [2018] (24) | 2015-2017 | 42 / 41 | 61 / 60 | 38 / 39 | Tamsulosin, 0.4 mg qd | None | 7 days | None | Proximal ureter; renal | Range not reported  (mean 11.0 mm***) | Single |
| Mohey [2018] (25) | 2015-2017 | 62 / 65 | 38 / 40 | 63 / 60 | Silodosin,  8 mg qd | Placebo,  qd | 10 days | None | Distal ureter | ≥ 10 mm  (mean 12.8 mm) | Single |
| Nadeem [2021] (26) | 2018-2019 | 75 / 75 | 35 / 35 *** | ⎯ | Tamsulosin, 0.4 mg bd | None | 7 days | None | Distal/mid ureter | 8-15 mm  (mean 13.5 mm) | Single |
| Rashahmadi [2018] (27) | 2013-2014 | 58 / 62 | 38 / 42 | 74 / 71 | Tamsulosin, 0.4 mg, bd | Placebo,  bd | 1 day | None | Ureter (varied) | Range not reported  (mean 10.1 mm***) | Single (76%)  Multiple (24%) |
| Shalaby [2022] (28) | 2017-2019 | 40 / 40 | 35 / 38 | 70 / 73 | Tamsulosin, 0.4 mg, qd | Placebo,  qd | 56 days | None | Distal ureter | 10-15 mm  (mean 10.8 mm) | Single |
| Tawfeek [2020] (29) | 2017-2018 | 58 / 62 | 39 / 38 | 63 / 63 *** | Tamsulosin, 0.4 mg qd | Placebo,  qd | 7 days | 14 days | Distal ureter | 5-20 mm  (mean 11.4 mm) | Single |

*Values reported for alpha blocker group / control group.

**Treatment groups combined because of a single control group.

***Estimated values.

**Abbreviations:** URS=ureteroscopy.

**Table S3. Definitions and modalities used to determine stone-free status**

| **Study** | **Stone-free definition** | **Diagnostic test** | **Diagnostic test timing** |
| --- | --- | --- | --- |
| Abdelaziz [2017] (15) | No residual stone fragment >2 mm diameter | KUB, ultrasound | 1 day, 2 weeks |
| Ahmed [2017] (16) | No residual stone fragment >2 mm diameter | KUB, ultrasound, CT (4 weeks only) | 2, 4, & 8 weeks |
| Aydin [2018] (17) | Not reported | KUB, ultrasound, CT | 1 month |
| Bayar [2019] (18) | Not reported | CT | 4 weeks |
| Bhattar [2017] (19) | Not reported | Not reported | Not reported |
| Bhattar [2018] (20) | Not reported | KUB, ultrasound | Postoperatively |
| Demir [2022] (21) | No residual stone fragment >3 mm diameter | KUB | Postoperatively |
| Ketabchi [2014] (22) | No residual fragments or signs of hydronephrosis | KUB, ultrasound | 1 day, 1 week, 2 weeks |
| Kim [2022] (23) | No residual stone fragment >2 mm diameter | CT | 3 months |
| Koo [2018] (24) | Not reported | Ultrasound | 3 months |
| Mohey [2018] (25) | No residual stone fragment >2 mm diameter or signs of hydronephrosis | KUB, ultrasound | 1-2 days, 4 weeks |
| Nadeem [2021] (26) | Not reported | KUB | 2 weeks |
| Rashahmadi [2018] (27) | Not reported | Not reported | Not reported |
| Shalaby [2022] (28) | Not reported | Ultrasound, KUB, or CT (8 weeks or after self-reported expulsion) | 1-8 weeks |
| Tawfeek [2020] (29) | Residual stone or backpressure change | CT | 1 month |

**Table S4. Association of patient- and study-factors on the risk of residual stone and complications with alpha-blocker therapy before ureteroscopy.***

| **Variable** | **Residual Stone** | | **Complications** | |
| --- | --- | --- | --- | --- |
|  | **z-value**** | **p-value** | **z-value**** | **p-value** |
| Stone location (qualitative) |  |  |  |  |
| Renal / proximal ureter vs. distal / mid ureter | 1.93 | 0.05 | 0.68 | 0.50 |
| Ureteral (varied) vs. distal / mid ureter | 0.89 | 0.37 | -0.89 | 0.37 |
| Stone location (% distal stones) | -1.57 | 0.12 | -0.42 | 0.67 |
| Stone size (mm) | -0.33 | 0.74 | -0.78 | 0.43 |
| Age (years) | 1.53 | 0.13 | 0.85 | 0.39 |
| Female sex (%) | 1.68 | 0.09 | 1.00 | 0.32 |
| Silodosin vs. tamsulosin | 1.31 | 0.19 | -0.01 | 0.99 |
| No placebo vs. placebo | 1.75 | 0.08 | 0.40 | 0.69 |
| Treatment duration (days) | -0.23 | 0.82 | -0.79 | 0.43 |

*Results derived from random effects meta-regression.

**Positive z-value and p<0.05 indicate the variable reduced the overall benefit of alpha-blockers; negative z-value and p<0.05 indicate the variable improved the overall benefit of alpha-blockers.

**Table S5. Grading of Recommendations Assessment, Development and Evaluation (GRADE) certainty of evidence.**

| **Outcome** | **Quality Assessment** | | | | | **No. of patients** | | **Effect**  **size** | **Certainty***** |
| --- | --- | --- | --- | --- | --- | --- | --- | --- | --- |
|  | **RoB** | **Inconsistency** | **Indirectness** | **Imprecision** | **Publication bias** | **Alpha**  **blockers** | **Controls** |  |  |
| Ureteral  dilatation | Low | Moderate | Low | Low | Low | 420 | 383 | RR=0.48  95% CI: 0.30, 0.75 | Moderate |
| Stone access  failure | Low | Low | Low | Low | Low | 368 | 332 | RR=0.36  95% CI: 0.23, 0.57 | High |
| Procedure  time (min) | Low | Moderate | Low | Low | Low | 609 | 572 | MD=-6  95% CI: -8, -3 | Moderate |
| Residual stone,  unadjusted | Moderate | Low | Low | Low | Low | 722 | 687 | RR=0.44  95% CI: 0.33, 0.60 | Moderate |
| Residual stone,  adjusted | Low | Low | Low | Low | Low | 742 | 696 | RR=0.52  95% CI: 0.40, 0.68 | High |
| Hospital  stay (d) | Moderate | Low | Low | Low | Low | 342 | 347 | MD=-0.3  95% CI: -0.4, -0.1 | Moderate |
| Complications,  any | Low | Low | Low | Low | Low | 806 | 770 | RR=0.46  95% CI: 0.35, 0.59 | High |
| Complications,  serious | Low | Moderate | Low | Moderate | Low | 651 | 658 | RR=0.66  95% CI: 0.26, 1.64 | Moderate |

*Results derived from random effects meta-regression.

**Positive z-value and p<0.05 indicate the variable reduced the overall benefit of alpha-blockers; negative z-value and p<0.05 indicate the variable improved the overall benefit of alpha-blockers.

***Evidence certainty graded as high, moderate, low, or very low. High=we are very confident that the true effect lies close to that of the estimate of the effect; Moderate=we are moderately confident in the effect estimate; the true effect is likely to be close to the estimate of the effect, but there is a possibility that it is substantially different; Low=our confidence in the effect estimate is limited; the true effect may be substantially different from the estimate of the effect; Very low certainty=we have very little confidence in the effect estimate; the true effect is likely to be substantially different from the estimate of effect.

**Abbreviations:** CI=confidence interval; MD=mean difference; RoB=risk of bias; RR=risk ratio.


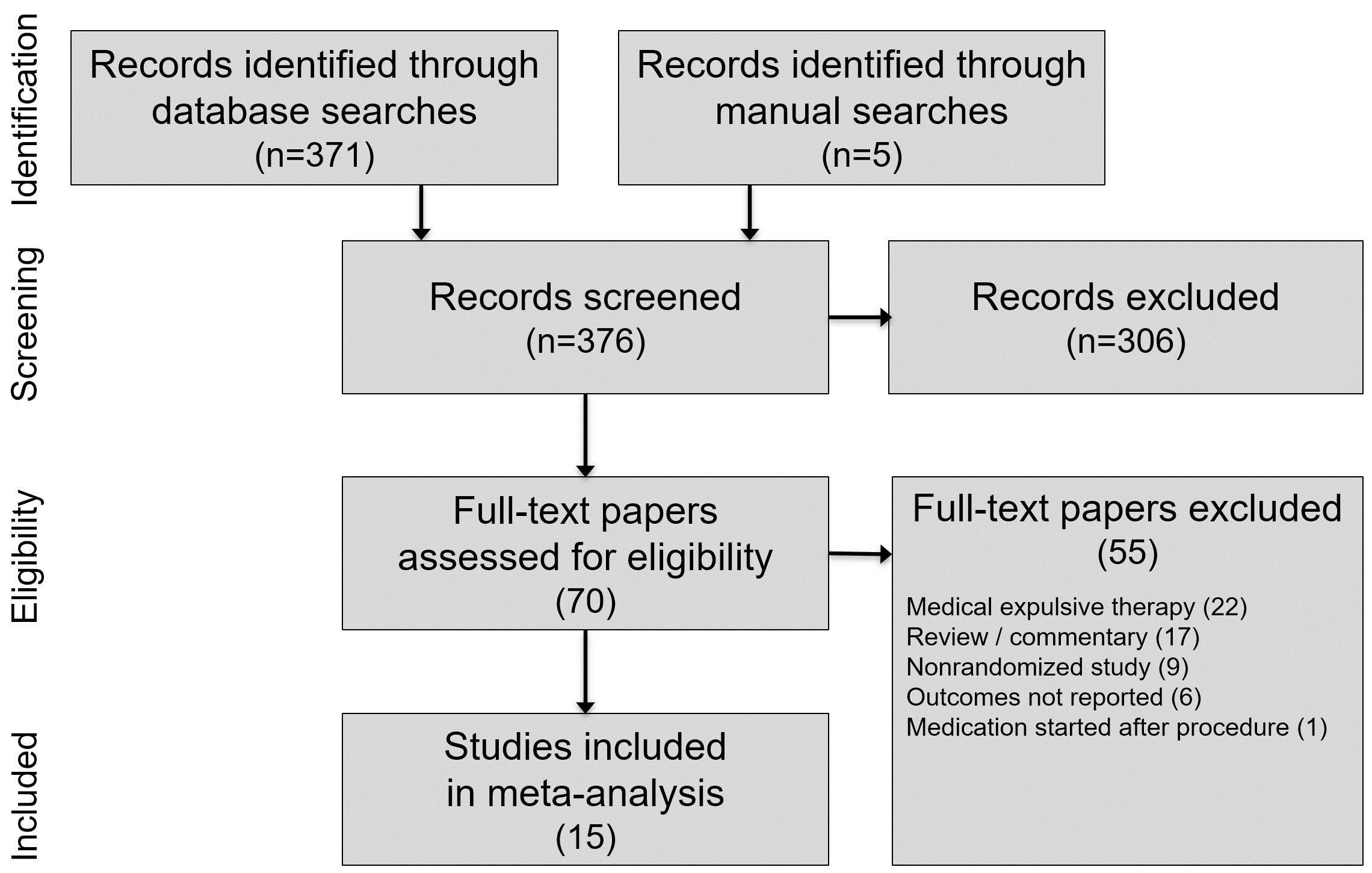


**Figure S1. PRISMA flow diagram.**


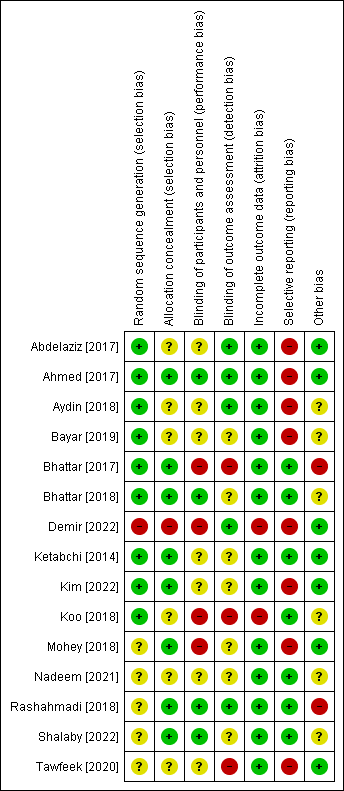


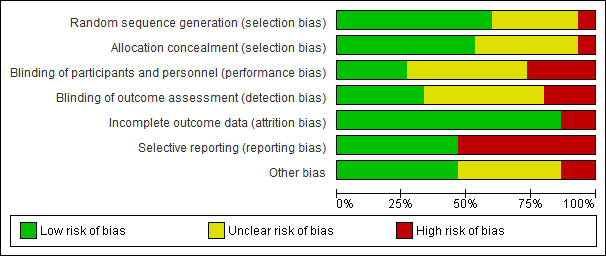


**Figure S2. Risk of bias summary**. Review authors' judgments about each risk of bias item for each included study (top) and presented as percentages across all included studies (bottom).


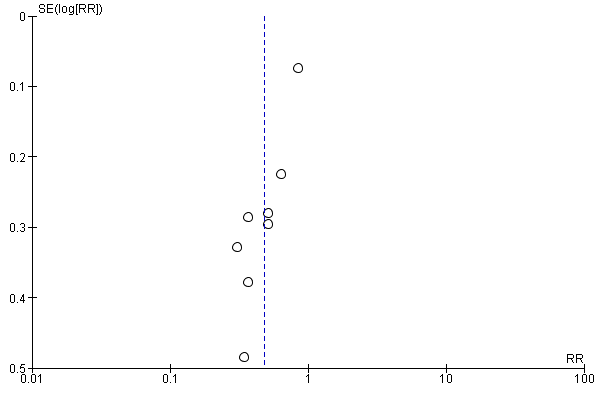


**Figure S3. Funnel plot of alpha-blocker therapy before ureteroscopy on the risk of requiring ureteral dilatation.**

**Abbreviations:** RR=risk ratio; SE=standard error.


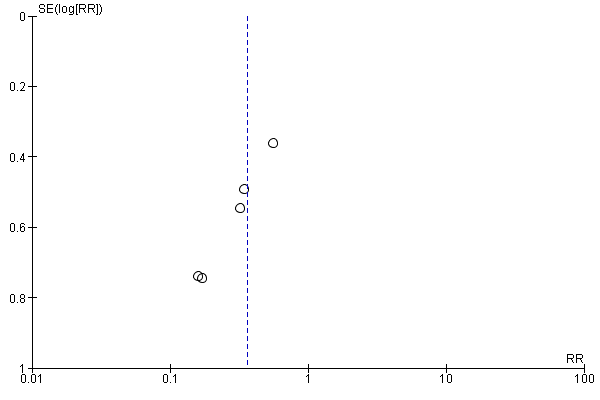


**Figure S4. Funnel plot of alpha-blocker therapy before ureteroscopy on the risk of stone access failure.**

**Abbreviations:** RR=risk ratio; SE=standard error.


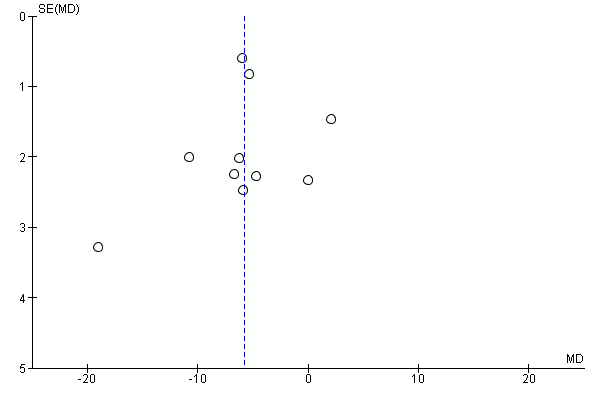


**Figure S5. Funnel plot of alpha-blocker therapy before ureteroscopy on procedure time.** Values reported in minutes.

**Abbreviations:** MD=mean difference; SE=standard error.


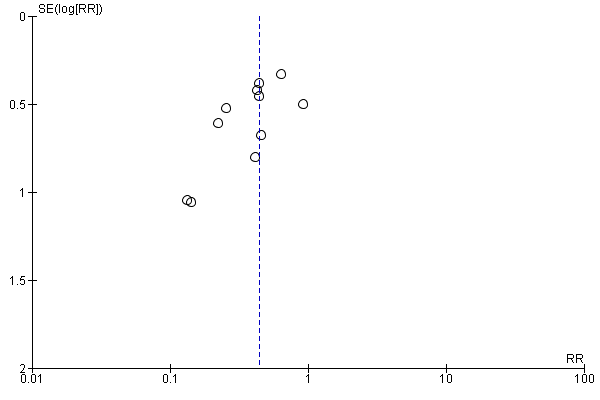


**Figure S6. Funnel plot of alpha-blocker therapy before ureteroscopy on the risk of residual stone.**

**Abbreviations:** RR=risk ratio; SE=standard error.


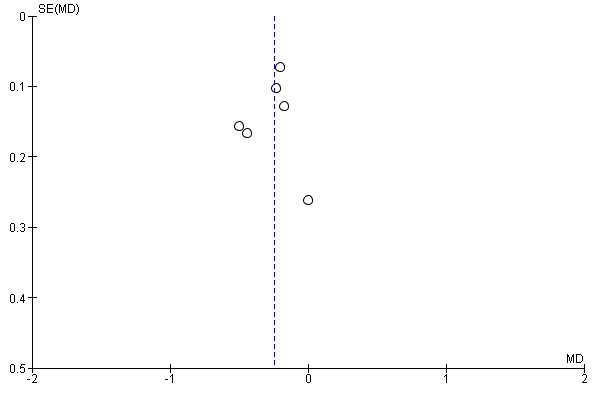


**Figure S7. Funnel plot of alpha-blocker therapy before ureteroscopy on hospital stay.** Values reported in days.

**Abbreviations:** MD=mean difference; SE=standard error.


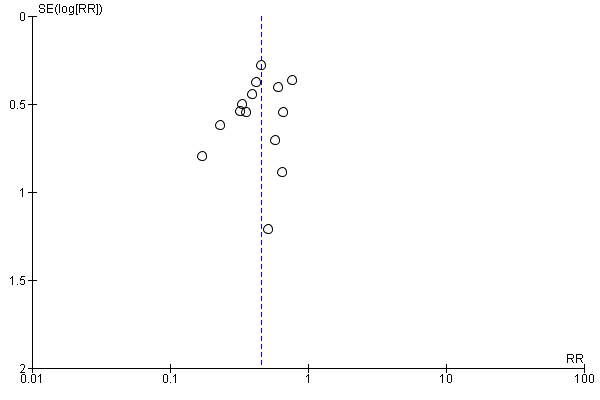


**Figure S8. Funnel plot of alpha-blocker therapy before ureteroscopy on the risk of complications.**

**Abbreviations:** RR=risk ratio; SE=standard error.


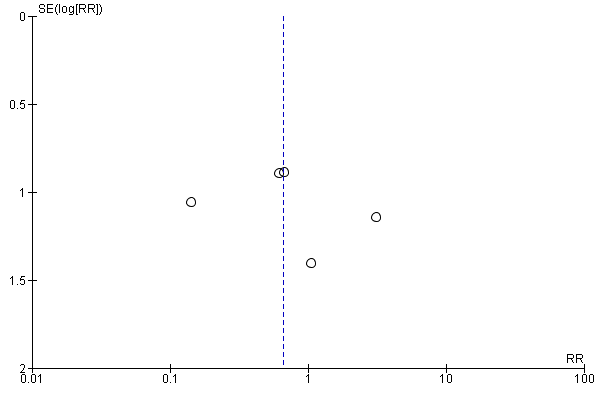


**Figure S9. Funnel plot of alpha-blocker therapy before ureteroscopy on the risk of serious complications.**

**Abbreviations:** RR=risk ratio; SE=standard error.


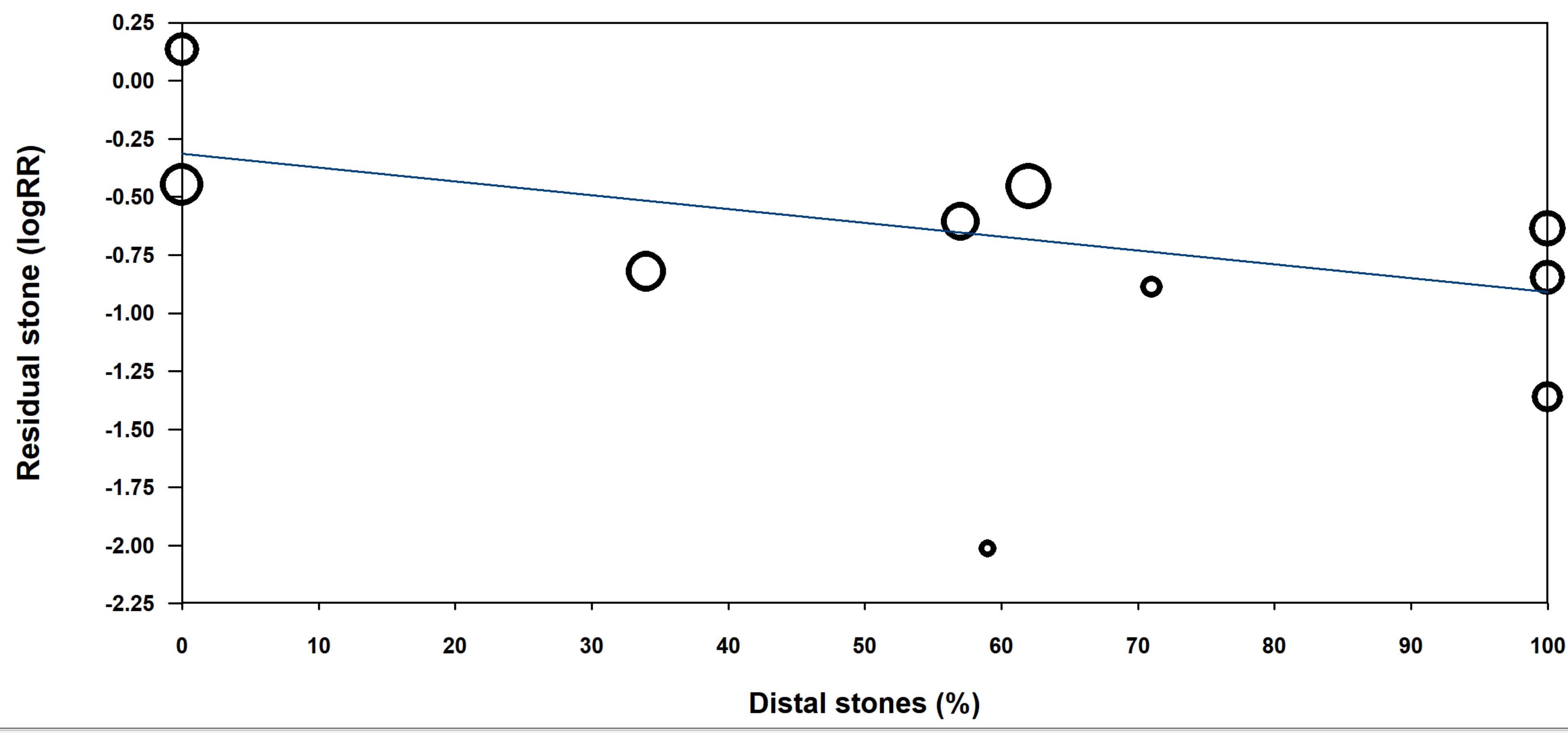


**Figure S10. Bubble plot of the association between the log risk ratio of residual stones and stone location.** Open circles represent values of individual studies where the circle size is proportional to the study weight in the random-effects model. The red line represents the regression line of best fit. A log risk ratio (logRR) value of 0 indicates no effect of alpha-blockers; negative values indicate lower risk of residual stone with alpha-blockers.
